# Factor X Concentrate Treatment Schedule and Dosing in Acquired FX Deficiency

**DOI:** 10.3390/hematolrep17020010

**Published:** 2025-02-21

**Authors:** Andrew Ross, Rebecca J. Shaw, Louise Garth, Cathy Farrelly

**Affiliations:** 1 Liverpool University Hospital NHS Foundation Trust, Liverpool L7 8XP, UK; r.shaw3@nhs.net (R.J.S.); louise.garth@nhs.net (L.G.); cathy.farrelly@liverpoolft.nhs.uk (C.F.); 2 Department of Clinical Infection, Microbiology and Immunology, Institute of Infection, Veterinary and Ecological Sciences, Leahurst Campus, University of Liverpool, Liverpool CH64 7TEK, UK

**Keywords:** factor X, Coagadex, amyloidosis

## Abstract

Background: Acquired factor X (FX) deficiency is a rare condition that can cause life threatening bleeding. Here we outline a successful management strategy for gastrointestinal bleeding (GI) using human FX concentrate. Case description: A 61-year-old male presented with upper GI bleeding and a prolonged prothrombin time. Investigations demonstrated an acquired FX deficiency (determined to be secondary to AL amyloidosis). Results: Treatment with FX concentrate to maintain trough FX levels >20% resulted in successful cessation of bleeding symptoms, and levels >50% facilitated urgent invasive procedures. Conclusions: This case report adds valuable insight into the management of this rare condition, and how best to utilize FX concentrates in acquired FX deficiency.

## 1. Introduction

Factor X (FX) deficiency is a rare inherited disorder leading to a variable bleeding phenotype, from mucosal bleeding to life threatening haemorrhages [1,2,3]. In the acquired form, the most common cause is primary amyloidosis (AL), with deposition of abnormal insoluble proteins due to excessive release of light chains from an expanded plasma cell clone [4]. FX deficiency occurs in 8.7–14% of AL amyloid [3,5]. Circulating FX is adsorbed onto amyloid fibrils causing a significant reduction in half-life and a quantitative deficiency.

There is little evidence supporting the management of these cases. Historically, factor replacement involved using fresh frozen plasma (FFP), prothrombin complex concentrate (PCC) and recombinant activated factor VII (rVIIa) [6]; complications with fluid overload and thrombotic events are reported, as well as treatment often being unsuccessful due to rapid removal of FX from the circulation. Recent years have seen the development of FX concentrates for inherited FX deficiency. However, experience of such concentrates in acquired FX deficiency is very limited, with only three such cases in the literature [7,8]. Here, we present a case highlighting the utility of FX concentrate in the management of bleeding in acquired FX deficiency and demonstrate an effective once daily dosing regimen.

## 2. Case Details

### 2.1. Patient Information

A 61-year-old male presented with severe abdominal pain and melaena. He reported a 6-week history of weight loss, ankle swelling and macroglossia. A recent gastroscopy for persistent reflux was reported as showing Barrett’s oesophagus, but there were no other comorbidities of note. There were no regular medications and no family history.

### 2.2. Clinical Findings and Diagnostic Assessment

Initial presenting symptoms and investigations suggested a significant upper gastro-intestinal bleed. An OGD was planned, but the INR was 2.9, in the absence of anticoagulation. Subsequently, the patient’s haemoglobin dropped to 83 g/L (reference range, 133–167 g/L) and he became haemodynamically unstable. Prothrombin time (PT) was elevated at 36.9 s (range 9–13 s) with a normal activated partial thromboplastin time (aPTT) of 29.7 s (range 29–30 s) and fibrinogen of 4.58 g/L (range 1.5–3.5 g/L). Historical coagulation screens were normal. Mixing studies showed correction of PT to 15.0 s, with no change on incubation. FX activity was quantified at 7.0% (range 50–150%). The FX assay was performed using a modified prothrombin time assay on the ACLTOP coagulation analyser. Patient plasma was diluted and FX-deficient plasma added; the PT was then measured. FX concentration (% activity) was calculated by using the measured PT, which is proportional to the FX activity, and plotted against a calibration curve. Other factor activity assays included factor II (92%, [range 50–150%]), factor V (173%, range [50–150%]) and factor VII (115%, [range 50–150%]).

### 2.3. Therapeutic Intervention

A diagnosis of amyloidosis was suspected, with acquired FX deficiency. Initially, 10 mg IV vitamin K and prothrombin complex concentrate (Beriplex, 4000 units, CSL Behring, Marburg, Germany) were administered, which led to an improvement in PT (23.8 s), and FX (18%), but with little change in bleeding symptoms. The patient was transferred urgently to a haemophilia comprehensive care centre.

**Figure 1:**
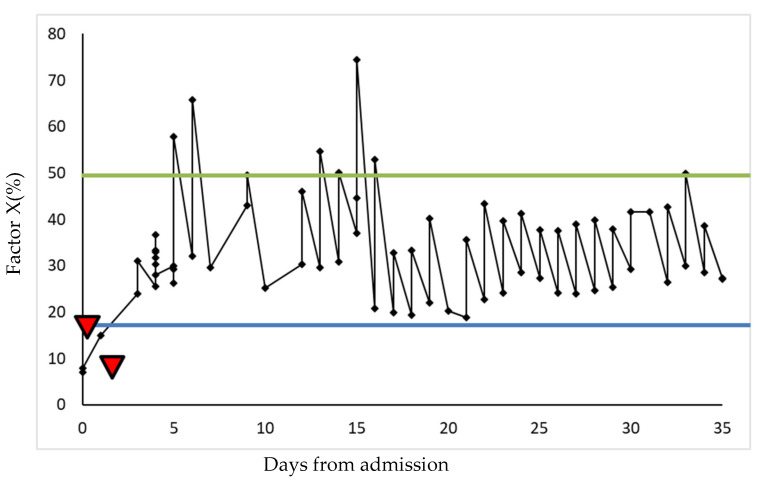
Trends in FX activity throughout hospital admission. Showing factor X level (%) at different time points during admission, pre- and post-FX concentrate treatment. FX concentrate was initially dosed at [body weight × desired factor 10 rise × 0.5]. Doses were increased by 100% until the desired factor X levels were achieved. Maintaining peak and trough factor X levels above 20% (blue line) resulted in significant improvement in bleeding episodes (denoted by red triangles). A threshold of 50% was used to facilitate interventions (green line), with no post-procedural haemorrhage reported.

Coagadex (human coagulation FX, Bio Products Laboratory Limited, Borehamwood, UK) was commenced according to the dosing for inherited FX deficiency (body weight × desired FX rise × 0.5) once daily. Post-dose levels taken 30 min after administration of FX concentrate demonstrated an FX level of 24.0% and some improvement in bleeding. However, to achieve haemostasis, subsequent doses were doubled (calculated by body weight × desired FX rise), leading to a post-dose FX of 36.7%. An emergency gastroscopy showed severe diffuse oesophageal bleeding with a friable mucosa; haemostatic clips and adrenaline were utilised. To facilitate this procedure, FX concentrate was dosed to achieve FX of >50% with no significant post-procedural bleeding identified (see Figure 1).

### 2.4. Follow-Up and Outcomes

Over the next two weeks, the patient’s symptoms remained stable other than mild but persistent bleeding from longstanding haemorrhoids. This was managed conservatively with daily FX concentrate, and the bleeding eventually subsided. There were no thrombotic complications during treatment.

**Figure 2:**
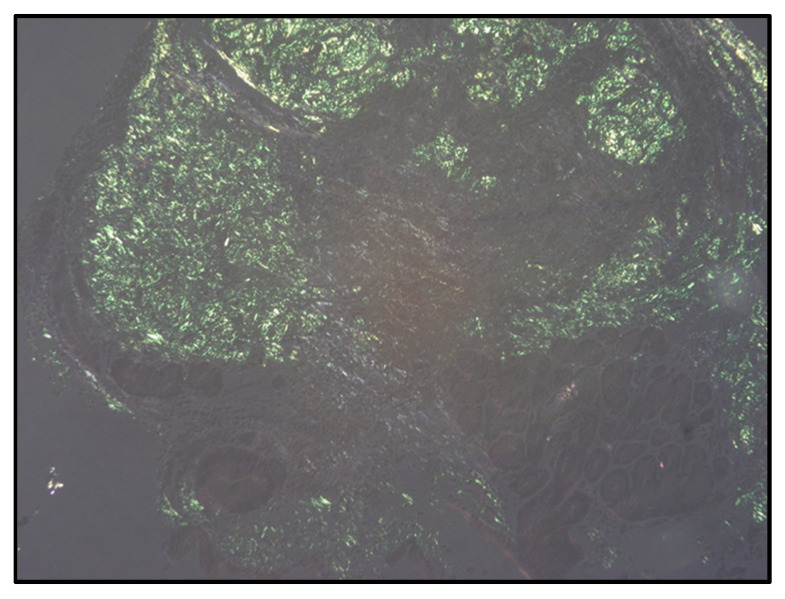
Congo red stain of gastric biopsy. Gastric biopsy from patient with Congo red stain showing apple green bi-refringence, diagnostic of amyloid infiltration.

A diagnosis of AL amyloidosis with gastric, renal and cardiac involvement was confirmed (kappa:lambda light chain ratio of 484, [kappa light chain 2372 mg/L], BNP 26,409 mg/L, urine albumin–creatinine ratio 10.4 mg/mmol). Bone marrow aspirate identified 9% plasma cells, but the trephine sample was inadequate. To avoid repeated invasive procedures, the historical gastroscopy biopsies were re-reviewed and sent for Congo red staining, which revealed classical apple-green birefringence under polarised light (Figure 2). The patient was commenced on velcade, thalidomide and dexamethasone chemotherapy, but did not tolerate treatment and was placed on palliative care shortly afterwards.

## 3. Discussion

Acquired FX deficiency is a rare complication of amyloid fibril deposition secondary to an underlying plasma cell neoplasm [3]. In inherited disease, bleeding symptoms typically occur below FX levels of 10%, but the threshold for bleeding in acquired disease appears lower, with one study demonstrating an increased risk of haemorrhage below 25% [3]. This may be due to other causes of haemostatic defects in AL amyloid, including small vessel fragility due to amyloid infiltration, deficiencies of other coagulation factors, abnormal fibrin polymerization, vitamin K deficiency and thrombocytopenia/dysfunctional platelets [4]. The major underlying mechanism is postulated to be adsorption of FX by amyloid deposits. Additionally, the macrophage scavenger receptor class A member 1 (SR-A1) binds FX, leading to its internalisation and degradation [9]. This action is opposed by pentaxin-2, which forms a complex with FX/SR-A1 preventing internalisation. Amyloid fibrils may lead to a reduction in pentaxin-2 leading to increased FX depletion [9].

FFP, PCC and rVIIa therapies are associated with thrombotic complications as well as fluid overload, particularly problematic in AL amyloid patients with frailty and cardiac compromise. The availability of high-purity FX concentrates has seen a change in management for inherited FX deficiency, but there are limited data on the use in acquired deficiency.

Coagadex is a single-factor concentrate licenced for the treatment of inherited FX deficiency. Here, we demonstrate the effective use of FX concentrate to control bleeding symptoms and effectively facilitate invasive procedures. Though the literature suggests that FX < 25% confers increased bleeding risk, we demonstrate that trough levels >20% caused cessation of bleeding symptoms. Furthermore, the patient did not develop thrombotic complications and, despite the significant cardiac amyloid burden, did not develop pulmonary oedema. One report (*n* = 2) demonstrated effective bleeding control with levels >15% but required more frequent dosing to achieve this [7]. In this case, by increasing the dose of FX concentrate (by 100%), we were able to administer a once daily dosing regimen to maintain trough levels above the desired threshold of 20%. The variability between cases could reflect the heterogeneity of amyloid deposition and subsequent impact on factor levels.

Significant bleeding symptoms here were successfully managed with FX concentrate. Bleeding symptoms were controlled by maintaining FX levels >20% and invasive procedures facilitated at levels >50%, without bleeding complications. It is important to note that significantly higher doses of FX concentrate were required compared to the recommended dosing regimen for congenital FX deficiency. There is an argument for screening patients with AL amyloid for FX deficiency to pre-empt bleeding diatheses, particularly around invasive procedures. Further data are needed in this rare disease area to determine optimal management strategies with FX concentrates. We propose an individualised approach to FX concentrate dosing, threshold targets and frequency of administration.

## Data Availability

Data are contained within the article/available on request from the authors.

## References

[1] Menegatti M., Peyvandi F. (2009). Factor X deficiency. Semin. Thromb. Hemost..

[2] Mumford A.D., Ackroyd S., Alikhan R., Bowles L., Chowdary P., Grainger J., Mainwaring J., Mathias M., O’Connell N. (2014). Guideline for the diagnosis and management of the rare coagulation disorders: A United Kingdom Haemophilia Centre Doctors’ Organization guideline on behalf of the British Committee for Standards in Haematology. Br. J. Haematol..

[3] Peyvandi F., Auerswald G., Austin S.K., Liesner R., Kavakli K., Román M.T.Á., Millar C.M. (2021). Diagnosis, therapeutic advances, and key recommendations for the management of factor X deficiency. Blood Rev..

[4] Nicol M., Siguret V., Vergaro G., Aimo A., Emdin M., Dillinger J.G., Baudet M., Cohen-Solal A., Villesuzanne C., Harel S. (2022). Thromboembolism and bleeding in systemic amyloidosis: A review. ESC Heart Fail..

[5] Patel G., Hari P., Szabo A., Rein L., Kreuziger L.B., Chhabra S., Dhakal B., D’Souza A. (2017). Acquired Factor X Deficiency in Light Chain (AL) Amyloidosis Is Rare and Associated with Advanced Disease. Blood.

[6] Dejhansathit S., Suvannasankha A. (2019). Acquired Factor X Deficiency in Patients With Primary Light Chain Amyloidosis. J. Investig. Med. High Impact Case Rep..

[7] Mahmood S., Blundell J., Drebes A., Hawkins P.N., Wechalekar A.D. (2014). Utility of factor X concentrate for the treatment of acquired factor X deficiency in systemic light-chain amyloidosis. Blood.

[8] Duncan E.M., Cole J., Clarkson A.R., Lloyd J.V. (1999). Poor recovery and short survival of infused factor X in a case of acquired factor X deficiency and amyloidosis. Thromb. Haemost..

[9] Muczynski V., Aymé G., Regnault V., Vasse M., Borgel D., Legendre P., Bazaa A., Harel A., Loubière C., Lenting P.J. (2017). Complex formation with pentraxin-2 regulates factor X plasma levels and macrophage interactions. Blood.

